# Exploring the Evolution of Sleep Patterns From Infancy to Adolescence

**DOI:** 10.7759/cureus.64759

**Published:** 2024-07-17

**Authors:** Paula Goel, Ashish Goel

**Affiliations:** 1 Pediatrics, Fayth Clinic, Mumbai, IND; 2 Cardiology, Fayth Clinic, Mumbai, IND

**Keywords:** adolescent medicine, development of brain function, child and adolescent, infant sleep, school sleep policies, childhood sleep, sleep pattern

## Abstract

Sleep is a critical component of healthy development, particularly during the formative years from infancy through adolescence. Sleep undergoes continuous change throughout life characterized by frequent awakenings and a high proportion of rapid eye movement (REM) sleep during infancy, changes in sleep architecture, an increase in non-rapid eye movement (NREM) sleep during adolescence, and an eventual decrease in REM sleep in old age. Adequate sleep is therefore essential for cognitive development, especially between ages 10 and 16. Sleep deprivation may negatively affect academic performance, attention regulation, and emotional well-being. Biological factors, such as hormonal changes during puberty, significantly influence sleep patterns, leading to later bedtimes and a tendency for chronic sleep deprivation in adolescents. Environmental factors, including light exposure and screen time, also play a critical role in regulating sleep. This paper examines the evolution of sleep patterns across infancy and adolescence, describing changes in sleep architecture, timing, and regulation. The influence of biological, environmental, and socio-cultural factors on sleep is explored, highlighting how these factors collectively shape sleep behaviors and health outcomes. It also addresses the profound role sleep plays in cognitive development, brain maturation, and emotional well-being. The importance of understanding sleep patterns and their developmental trajectories to address sleep-related issues is emphasized. Promoting healthy sleep from an early age can enhance cognitive and emotional outcomes, contributing to better academic performance and overall well-being in children and adolescents. The findings advocate for further standardized sleep intervention programs globally to prioritize sleep health and support optimal development.

## Introduction and background

Sleep is a fundamental aspect of human development. The consistent evolution of sleep patterns, especially from infancy to adolescence, reflects the maturation of the central nervous system and physiological processes. Understanding these changes is key to identifying normal developmental trajectories and detecting deviations that may indicate health or behavioral issues. Over the past decade, research has advanced our understanding of sleep’s pivotal role in cognitive development.

Infancy and early childhood are marked by unique changes in sleep physiology and patterns as children transition from biphasic (naps and overnight sleep) to monophasic (primarily overnight sleep) sleep [1]. Naps in young children enhance learning through sleep-dependent memory consolidation [2]. Changes in sleep macrostructure (organization of sleep stages) and microstructure (EEG characteristics) accompany brain maturation, impacting this sleep transition [3].

From childhood to adolescence, sleep architecture, duration, and patterns change dramatically, with individual differences playing a significant role in cognitive and brain development. Vigilant attention, a core cognitive function, also evolves, showing rapid improvement between ages 10 and 16, moderate improvement through adulthood, and decline in later adulthood [4]. Understanding these developmental shifts is crucial for promoting optimal sleep health and addressing sleep-related issues in children and adolescents. This narrative review studies sleep and brain development across childhood, emphasizing the importance of healthy sleep for cognitive development. We highlight the relationship between brain maturation and early sleep patterns, which can inform how sleep opportunities are structured in school and home settings [5].

## Review

Sleep changes across early development

Sleep Architecture

Infancy: Sleep is divided into quiet sleep (QS) and active sleep (AS), precursors to non-rapid eye movement (NREM) and rapid eye movement (REM) sleep, respectively. QS and AS evolve into distinct NREM and REM stages as EEG patterns become more pronounced. Infants have a sleep cycle of approximately 60 minutes, which extends to 90 minutes in adults [6].

Early childhood (three to five years): Sleep transitions from biphasic (midday nap and overnight sleep) to monophasic (overnight sleep). NREM sleep comprises three stages: NREM stage 1: reduction in alpha activity (8-13 Hz). NREM stage 2: characterized by sleep spindles (short bursts of 11-16 Hz activity) and K-complexes (high-amplitude waves). NREM stage 3: Slow-wave sleep (SWS) with low-frequency cortical oscillations (0.4-4 Hz) [7].

Middle childhood to adolescence: Sleep architecture continues to evolve with reductions in SWS and increases in NREM Stage 2. REM sleep decreases from 50% in infancy to about 20% by adulthood. Sleep spindle density and amplitude peak in early childhood and adjust across the lifespan [1,8].

Sleep Timing and Regulation

Sleep is regulated by circadian rhythms (Process C) and homeostatic sleep pressure (Process S). Circadian rhythms align sleep with environmental light/dark cycles, while homeostatic pressure drives sleep needs. Young children accumulate sleep pressure rapidly, necessitating naps to dissipate this pressure. Napping supports learning and memory, with sleep spindles and SWS playing critical roles [2,3].

Infancy (zero to two years): Characterized by frequent awakenings, short sleep cycles, and predominant REM sleep. Newborns sleep 16-17 hours per day in short bouts. Nighttime sleep duration increases with age, and naps become more consolidated [9].

Early childhood (three to five years): Further consolidation of sleep patterns, with most children sleeping 10-12 hours per night plus a daytime nap. REM sleep comprises 20-25% of total sleep time. Bedtime routines facilitate sleep onset and maintenance [10].

Middle childhood (6-12 years): Total sleep duration decreases, and daytime napping becomes rare. School schedules influence sleep timing, and sleep disturbances like nightmares and sleepwalking may emerge [11,12].

Adolescence (13-18 years): Marked by later bedtimes and irregular sleep schedules due to biological, psychological, and social factors. Sleep duration decreases, with many adolescents experiencing chronic sleep deprivation. REM sleep reaches adult levels, and SWS remains vital for cognitive and emotional functions [9,13,14].

Factors influencing sleep patterns

Biological maturation is a critical factor in the evolution of sleep patterns. Genetic predispositions determine individual sleep needs and circadian rhythms, while hormonal changes during puberty significantly alter sleep architecture and timing. The combination of genetics and environment shapes sleep behaviors and overall sleep health [15-17]. During adolescence, the onset of puberty triggers significant hormonal changes, particularly in the production of melatonin by the pineal gland, which influences sleep-wake cycles. The delay in melatonin secretion during puberty leads to a natural tendency for adolescents to stay up later and wake up later, contributing to the phenomenon known as "delayed sleep phase syndrome" [5]. This shift in circadian rhythms often conflicts with early school start times, resulting in chronic sleep deprivation among teenagers [14].

Hormonal changes during puberty are not limited to melatonin but also include significant alterations in other hormones such as growth hormone, cortisol, and sex steroids (testosterone and estrogen). These hormones play critical roles in regulating various bodily functions, including sleep [8]. For instance, growth hormone, which is crucial for physical development, is predominantly secreted during deep sleep stages, particularly during puberty when rapid growth occurs. Any disruption in sleep patterns can thus impact growth hormone secretion and consequently physical growth and development [8]. Cortisol, a hormone associated with stress response, follows a diurnal pattern with levels peaking in the early morning and declining throughout the day. During adolescence, the cortisol awakening response can be heightened, contributing to difficulties in falling asleep and waking up early. Increased cortisol levels can also lead to fragmented sleep and reduced overall sleep quality, further complicating the sleep architecture during this critical developmental period. Sex steroids such as testosterone and estradiol also influence sleep patterns. Testosterone levels, which increase significantly during puberty, have been linked to changes in sleep architecture, including alterations in REM sleep [18]. Estradiol, which fluctuates with the menstrual cycle in females, can affect sleep quality and patterns, with many adolescent girls experiencing sleep disturbances related to hormonal changes during their menstrual cycles.

Environmental factors, including light exposure, noise, and temperature, further play a role in regulating sleep patterns. Excessive screen time, particularly before bedtime, can disrupt circadian rhythms and reduce sleep quality. Blue light emitted from screens inhibits melatonin production, delaying sleep onset and reducing overall sleep duration [19]. Exposure to natural light during the day helps to align circadian rhythms with the environmental light/dark cycle, promoting better sleep at night.

Parenting practices and cultural norms also influence sleep patterns. Consistent bedtime routines and sleep hygiene practices can facilitate healthy sleep habits in children. Cultural differences in sleep practices, such as co-sleeping or independent sleeping, impact sleep quality and patterns [10]. For example, cultures that emphasize communal sleeping arrangements may see different sleep patterns compared to those that prioritize independent sleeping. In some cultures, late bedtimes are more socially acceptable, while others emphasize early bedtimes and structured sleep schedules. These cultural differences can influence parental attitudes toward sleep and the enforcement of bedtime routines [10].

Socioeconomic status (SES) can further influence sleep patterns through factors such as access to a safe and quiet sleeping environment, parental work schedules, and educational demands. Children from lower SES backgrounds may experience more sleep disturbances due to environmental stressors, crowded living conditions, and irregular routines [17]. Economic constraints often limit access to resources that promote healthy sleep, such as comfortable mattresses, quiet sleeping environments, and rooms with adequate darkness and ventilation. Additionally, lower SES is often associated with neighborhoods that are noisier and less safe, which can lead to difficulties in falling and staying asleep. Parental work schedules in lower SES families can also affect children's sleep patterns. Parents who work multiple jobs or have irregular hours may struggle to enforce consistent bedtime routines. This lack of routine can result in insufficient sleep and poorer sleep quality. In contrast, higher SES families typically have more predictable work schedules, allowing for more structured and consistent bedtime routines, which are conducive to better sleep health. Educational demands also play a role in the relationship between SES and sleep patterns. Children from higher SES families often have access to better educational resources and environments that support healthy sleep habits, such as schools that understand the importance of adequate sleep and may have later start times. Conversely, children from lower SES backgrounds may face increased academic pressure and longer hours of study or work to support their families, leading to reduced sleep duration and quality.

Individual differences, such as temperament and personality, affect sleep patterns and behaviors. Children with higher anxiety levels may experience more sleep disturbances, while those with a more relaxed temperament may have fewer sleep issues. Understanding these individual differences can help tailor interventions to promote healthy sleep habits [16,17]. For instance, interventions for anxious children might include strategies to reduce bedtime anxiety, such as relaxation techniques or cognitive-behavioral approaches.

Vigilant attention and sleep

Vigilant attention, the ability to maintain focused and sustained concentration, improves significantly from childhood through adolescence, with rapid advancements between ages 10 to 16 and continued, albeit slower, improvements into adulthood. Functional neuroimaging studies have shown that brain activation during sustained attention tasks increases with age, reflecting the maturation of neural mechanisms underlying this cognitive function [4,20]. This development is vital for academic performance and daily activities, as attention serves as a fundamental cognitive skill necessary for learning and performing complex tasks.

Sleep deprivation in adolescents has profound effects on daytime functioning, school performance, and attention. Adolescents experiencing sleep deprivation often exhibit impaired cognitive performance, marked by increased lapses in attention and poorer overall school performance. The regulation of sleep involves both homeostatic processes, which drive the need for sleep, and circadian rhythms, which align sleep patterns with the day-night cycle. These processes significantly influence cognitive performance [21,22]. Sleep deprivation particularly impacts the prefrontal cortex, a brain region crucial for executive functions such as decision-making, impulse control, and attention regulation. As a result, sleep-deprived adolescents may engage in more risk-taking behaviors, experience mood swings, and face difficulties in academic settings [22-24].

The negative impact of sleep deprivation on academic performance is well-documented. Adolescents who do not receive sufficient sleep often struggle with concentration, memory retention, and problem-solving skills, leading to lower grades and academic achievement [23]. Chronic sleep deprivation can also exacerbate conditions like attention deficit hyperactivity disorder (ADHD), further hindering academic success. Recommended sleep durations are 10-13 hours for children aged three to five years, nine to 11 hours for children aged six to 13 years, and eight to 10 hours for adolescents aged 14-17 years. Despite sleep loss, older adolescents often perform better than younger children due to ongoing brain maturation that enhances higher-order cognitive functions [23]. Promoting healthy sleep habits, such as maintaining a consistent sleep schedule, reducing screen time before bed, and creating a conducive sleep environment, can help to improve sleep duration and quality.

Schools and policymakers play a pivotal role in fostering healthy sleep habits among children and adolescents. Research indicates that delaying school start times can lead to improved sleep duration, better academic performance, and enhanced overall well-being in adolescents [22]. Implementing sleep education programs can raise awareness about the importance of sleep and provide students with practical strategies to enhance their sleep habits.

The cognitive and emotional consequences of sleep deprivation extend beyond academic performance. Sleep-deprived adolescents are more likely to experience symptoms of depression, anxiety, and irritability. Sleep deprivation can impair emotional regulation, leading to increased sensitivity to stress and difficulties in managing emotions [22]. These emotional challenges can negatively impact social relationships and overall quality of life. 

The relationship between sleep and vigilant attention highlights the critical role of adequate sleep for cognitive functions. Adolescents suffering from sleep deprivation exhibit impaired cognitive performance, especially in tasks requiring sustained attention. This impairment is associated with decreased activation in brain regions responsible for attention and executive functions [21]. Research indicates that sufficient sleep enhances vigilant attention and overall cognitive performance. Adolescents who receive the recommended amount of sleep demonstrate better academic performance and fewer behavioral problems compared to their sleep-deprived peers. These findings emphasize the need for policies and interventions that promote healthy sleep habits among adolescents [23,24].

Relations between sleep and brain development

Sleep modulates brain activity during development. Cao et al. found that sleep changes after two to three years of age are mainly for neural repair and clearance, while earlier changes support neural reorganization and learning, especially during REM sleep [3]. Kocevska et al. showed that childhood sleep disturbances correlate with smaller gray matter volumes, particularly in the dorsolateral prefrontal cortex, although causality is uncertain [25].

REM sleep is vital for brain development, especially in the visual cortex and motor system [6,9]. Studies on animals show that deprivation of REM sleep delays critical developmental periods and affects neural plasticity. In human infants, increased retinal activity during REM sleep suggests its role in the maturation of the visual system [3,26]. Myoclonic twitches occurring during early REM sleep may support motor system development [9,5]. Recent research has also shown that REM sleep deprivation can impact long-term potentiation, a process essential for memory and learning [27,28].

NREM sleep, especially SWS, is important for synaptic homeostasis and cortical plasticity [29]. Sleep spindles in early development help organize cortical maps and support sensorimotor development [30]. SWS changes reflect cortical maturation, with delta wave amplitude peaking in early childhood and declining through adolescence [31,32]. The topography of sleep slow-wave activity (SWA) changes with age, shifting from posterior to frontal regions [1]. This shift parallels gray matter maturation and is predictive of motor skill development and neural connectivity patterns [1,33]. Variations in SWA localization indicate experience-dependent plasticity and may assist in diagnosing developmental disorders [29,34].

Nap patterns in children may relate to hippocampal development. Habitual nappers tend to have larger hippocampal volumes, suggesting less mature short-term memory storage [15,35]. Sleep disturbances in early childhood, particularly in autism spectrum disorder (ASD), are linked to atypical brain morphology [36]. The topography and coherence of SWA reflect neural network development, with delta power shifting from occipital to frontal regions in alignment with cortical maturation [31,37]. Recent research has identified a sleep EEG signature that indicates brain maturation processes, specifically the ratio of frontal to occipital SWA (F/O ratio). The F/O ratio in SWA helps quantify regional development and predict brain maturation [1]. Experience-dependent changes in SWA also indicate how different brain regions respond to learning [29,38].

Furthermore, sleep promotes the formation and maintenance of dendritic spines, which are critical for synaptic plasticity [39,40]. SWS is associated with synaptic pruning, a process essential for maintaining brain efficiency [41]. During adolescence, sleep modulates synaptic connections more significantly than in adulthood, emphasizing its role in learning and memory. Poor sleep has been linked to increased symptoms of depression, anxiety, emotional distress, and academic setbacks [42]. 

The relationship between sleep and brain development is evident in the structural and microstructural changes that occur during sleep. REM sleep facilitates neural plasticity and the maturation of the visual and motor systems, while NREM sleep supports synaptic homeostasis and cortical development. These processes are crucial for forming and maintaining neural connections, which underpin cognitive functions and learning [6,29,40]. The topography of SWA reflects the maturation of cortical regions. Shifts in SWA from posterior to frontal regions align with gray matter development, indicating that sleep patterns can provide insights into brain maturation. Additionally, sleep has been found to play a significant role in white matter development, therefore excess sleep variability may impair white matter maturation [43]. These patterns also predict motor skill development and neural connectivity, highlighting the importance of sleep for overall brain health [1,33,43].

Understanding and promoting sleep for healthy cognitive development

Studies indicate that sleep patterns and brain development are closely intertwined. Changes in sleep, particularly shifts in SWA, mirror cortical maturation, suggesting a mutual influence between sleep physiology and brain development [1,8]. The consolidation of sleep patterns, such as the transition from biphasic to monophasic sleep, reflects underlying changes in brain structure and function.

Napping plays a critical role in early cognitive development by helping to manage sleep pressure accumulated during the day. Missing a midday nap can impair learning, especially in young children who may rely on naps for memory consolidation. Research shows that habitual nappers might have less developed hippocampi, necessitating more frequent memory offloading through naps [15,35]. Napping also supports emotional regulation and reduces daytime sleepiness, contributing to better overall functioning.

Sleep disturbances in early childhood are linked to structural brain changes and neurodevelopmental disorders such as attention deficit hyperactive disorder (ADHD) and ASD. For example, children with ADHD show less mature SWA distribution, while those with ASD exhibit atypical sleep EEG patterns. These findings highlight the potential of sleep EEG markers for early diagnosis of developmental disorders [21,23,44,45]. Furthermore, early intervention to address sleep disturbances in these populations can improve cognitive and behavioral outcomes.

In addition to neurodevelopmental disorders, physical developmental disorders, respiratory, gastrointestinal, and neurological disorders are also influenced by sleep. Physical developmental disorders, such as cerebral palsy, often involve sleep disturbances due to muscle spasticity and pain, affecting overall development and quality of life. Respiratory disorders like sleep apnea can cause fragmented sleep and hypoxia, leading to cognitive impairments and delayed growth. Gastrointestinal disorders, including gastroesophageal reflux disease (GERD), can disrupt sleep due to discomfort and pain, impacting development. Neurological disorders, such as epilepsy, frequently involve sleep disruptions, which can exacerbate seizure activity and further hinder cognitive and physical development.

Given the importance of naps in early development, educational policies should consider incorporating nap times into preschool schedules to support optimal cognitive growth. Longitudinal studies on sleep and brain development during the transition out of napping can provide deeper insights into the role of early sleep patterns [2,35]. Schools can also implement sleep education programs to teach children and parents about the importance of sleep and how to establish healthy sleep routines.

Interventions to improve sleep in children and adolescents can include behavioral strategies, environmental modifications, and policy changes. Behavioral strategies such as cognitive-behavioral therapy for insomnia (CBT-I) can help children develop better sleep habits and address issues such as bedtime resistance and anxiety [22]. Environmental modifications, such as reducing noise and light in the bedroom, can create a more conducive sleep environment. Policy changes, such as delaying school start times and limiting homework loads, can also support better sleep health [13,46].

Technology plays a dual role in sleep health. While excessive screen time can negatively impact sleep, technology can also be used to promote healthy sleep habits. For instance, apps that track sleep patterns and provide feedback can help individuals become more aware of their sleep behaviors and make necessary adjustments. Additionally, educational programs delivered through digital platforms can raise awareness about the importance of sleep and provide practical tips for improving sleep hygiene [23,47].

Family and community support are crucial for promoting healthy sleep habits in children and adolescents. Parents can model good sleep behaviors and establish consistent sleep routines. Communities can offer resources such as sleep workshops and support groups for parents and children. Schools and community organizations can collaborate to create environments that prioritize sleep health, such as providing quiet spaces for naps and reducing after-school activities that interfere with sleep [48].

Implications for health and well-being

Promoting healthy sleep habits from an early age is crucial for physical, emotional, and cognitive development. Sleep disruptions in childhood are linked to neurodevelopmental and mood disorders, obesity, and poor academic performance [9,13,22]. Insufficient sleep in adolescence increases risk-taking behaviors and mental health issues. Promoting healthy sleep habits early can thus mitigate these risks [9,24]. Adequate sleep is essential for physical health, supporting growth, immune function, and overall vitality. Sleep deprivation can weaken the immune system, making children more susceptible to illnesses. It also affects metabolic processes, contributing to weight gain and obesity. Ensuring that children get sufficient sleep can help prevent these health issues and promote overall well-being [19].

Sleep health is essential for overall well-being, with long-term implications for physical, cognitive, and emotional development. Insufficient or poor-quality sleep in childhood is associated with a range of adverse outcomes, including increased risk of neurodevelopmental and mood disorders. For instance, children with sleep disturbances are more likely to exhibit symptoms of ADHD and anxiety, which can persist into adolescence and adulthood if not addressed [9,13,22]. In adolescence, sleep deprivation is linked to risk-taking behaviors, such as substance abuse and unsafe driving, as well as mental health issues, including depression and anxiety. These findings emphasize the importance of promoting healthy sleep habits early in life to prevent these negative outcomes and support optimal development [9,24]. Additionally, sleep patterns and mental health are closely related, with poor sleep often correlating with increased anxiety and emotional concerns [20,42].

Children and adolescents who get sufficient sleep tend to perform better in school, with improved attention, memory, and problem-solving skills. Conversely, sleep-deprived students are more likely to struggle academically and exhibit behavioral issues [17,23,42,49]. Schools can play a pivotal role in promoting sleep health by adjusting start times, reducing homework burdens, and educating students about the importance of sleep. Children and adolescents who are well-rested are better able to manage their emotions and interact positively with peers and adults. Sleep deprivation can lead to irritability, mood swings, and social withdrawal, impacting relationships and overall social functioning [24]. Encouraging healthy sleep habits can improve social interactions and contribute to a more positive social environment [50].

The long-term implications of sleep health extend into adulthood. Establishing good sleep habits in childhood and adolescence sets the foundation for lifelong health and well-being. Adults who experience chronic sleep deprivation during their developmental years are at higher risk for a range of health issues, including cardiovascular disease, diabetes, and mental health disorders [19]. Promoting healthy sleep from an early age can help mitigate these risks and support a healthier adult population [51-54].

Sleep intervention programs for adolescents have demonstrated varying degrees of effectiveness in improving sleep behaviors, quality, and duration. These programs typically incorporate sleep hygiene education, cognitive-behavioral strategies, and relaxation techniques aimed at fostering better sleep habits. Studies indicate that such interventions can lead to significant improvements in sleep duration and quality, as well as a reduction in sleep onset latency [55-58]. School-based programs, in particular, have shown promise in increasing sleep knowledge and improving attitudes toward sleep, though translating these changes into significant behavioral improvements remains a challenge.

## Conclusions

Sleep patterns change significantly from infancy through adolescence, reflecting central nervous system maturation and environmental influences. Understanding these changes is essential for promoting sleep health and addressing sleep issues. The relationship between sleep and cognitive development highlights the critical role of sleep in shaping brain functions and behaviors. Adequate sleep is essential for maintaining high levels of attention and cognitive function, while sleep deprivation can severely impair these abilities. This indicates the need for targeted interventions to address sleep issues, particularly in adolescents, who are often at risk of chronic sleep deprivation due to various biological and social pressures.

Research on sleep intervention programs has been explored across various countries, which demonstrates that improved sleep among adolescents correlates with better functionality. Further trials of such programs conducted across geographies may be beneficial, especially if they include students from different academic systems. This may provide valuable insights as to how academic commitments and biological and societal factors may affect sleep and academic performance among school children in various regions. The subsequent findings could further inform policymakers, educators, and healthcare providers to prioritize sleep health to foster better cognitive development, academic performance, and overall well-being in children and adolescents. The promotion of healthy sleep habits, coupled with targeted interventions to address sleep-related issues, can significantly enhance cognitive and emotional outcomes, paving the way for healthier future generations.

## References

[1] Kurth S, Ringli M, Geiger A, LeBourgeois M, Jenni OG, Huber R (2010). Mapping of cortical activity in the first two decades of life: a high-density sleep electroencephalogram study. J Neurosci.

[2] Kurdziel L, Duclos K, Spencer RM (2013). Sleep spindles in midday naps enhance learning in preschool children. Proc Natl Acad Sci.

[3] Cao J, Herman AB, West GB, Poe G, Savage VM (2020). Unraveling why we sleep: quantitative analysis reveals abrupt transition from neural reorganization to repair in early development. Sci Adv.

[4] Barel E, Tzischinsky O (2022). The role of sleep patterns from childhood to adolescence in vigilant attention. Int J Environ Res Public Health.

[5] Dahl RE, Lewin DS (2002). Pathways to adolescent health sleep regulation and behavior. J Adolesc Health.

[6] Blumberg MS, Gall AJ, Todd WD (2014). The development of sleep-wake rhythms and the search for elemental circuits in the infant brain. Behav Neurosci.

[7] Tarokh L, Carskadon MA (2010). Developmental changes in the human sleep EEG during early adolescence. Sleep.

[8] Colrain IM, Baker FC (2011). Changes in sleep as a function of adolescent development. Neuropsychol Rev.

[9] Iglowstein I, Jenni OG, Molinari L, Largo RH (2003). Sleep duration from infancy to adolescence: reference values and generational trends. Pediatrics.

[10] Jenni OG, O'Connor BB (2005). Children's sleep: an interplay between culture and biology. Pediatrics.

[11] Leong RL, Lo JC, Chee MW (2021). Sleep-dependent prospective memory consolidation is impaired with aging. Sleep.

[12] Galland B, Spruyt K, Dawes P, McDowall PS, Elder D, Schaughency E (2015). Sleep disordered breathing and academic performance: a meta-analysis. Pediatrics.

[13] Paruthi S, Brooks LJ, D'Ambrosio C (2016). Consensus statement of the American Academy of Sleep Medicine on the recommended amount of sleep for healthy children: methodology and discussion. J Clin Sleep Med.

[14] Crowley SJ, Acebo C, Carskadon MA (2007). Sleep, circadian rhythms, and delayed phase in adolescence. Sleep Med.

[15] Riggins T, Spencer RM (2020). Habitual sleep is associated with both source memory and hippocampal subfield volume during early childhood. Sci Rep.

[16] Sadeh A, Gruber R, Raviv A (2002). Sleep, neurobehavioral functioning, and behavior problems in school-age children. Child Dev.

[17] Astill RG, Van der Heijden KB, Van Ijzendoorn MH, Van Someren EJ (2012). Sleep, cognition, and behavioral problems in school-age children: a century of research meta-analyzed. Psychol Bull.

[18] Wittert G (2014). The relationship between sleep disorders and testosterone in men. Asian J Androl.

[19] Hirshkowitz M, Whiton K, Albert SM (2015). National Sleep Foundation's sleep time duration recommendations: methodology and results summary. Sleep Health.

[20] Joo EY, Yoon CW, Koo DL, Kim D, Hong SB (2012). Adverse effects of 24 hours of sleep deprivation on cognition and stress hormones. J Clin Neurol.

[21] Page J, Lustenberger C, Frӧhlich F (2020). Nonrapid eye movement sleep and risk for autism spectrum disorder in early development: a topographical electroencephalogram pilot study. Brain Behav.

[22] Owens JA, Belon K, Moss P (2010). Impact of delaying school start time on adolescent sleep, mood, and behavior. Arch Pediatr Adolesc Med.

[23] Reddy R, Palmer CA, Jackson C, Farris SG, Alfano CA (2017). Impact of sleep restriction versus idealized sleep on emotional experience, reactivity and regulation in healthy adolescents. J Sleep Res.

[24] Short MA, Weber N (2018). Sleep duration and risk-taking in adolescents: a systematic review and meta-analysis. Sleep Med Rev.

[25] Kocevska D, Muetzel RL, Luik AI (2017). The developmental course of sleep disturbances across childhood relates to brain morphology at age 7: the Generation R study. Sleep.

[26] Peña M, Birch D (1999). The effect of sleep state on electroretinographic (ERG) activity during early human development. Early Hum Dev.

[27] Dumoulin Bridi MC, Aton SJ, Seibt J, Renouard L, Coleman T, Frank MG (2015). Rapid eye movement sleep promotes cortical plasticity in the developing brain. Sci Adv.

[28] Shaffery JP, Marks GA (2006). Rapid eye movement sleep deprivation modifies expression of long-term potentiation in visual cortex of immature rats. Neuroscience.

[29] Tononi G, Cirelli C (2006). Sleep function and synaptic homeostasis. Sleep Med Rev.

[30] Herrera CG, Tarokh L (2024). A thalamocortical perspective on sleep spindle alterations in neurodevelopmental disorders. Curr Sleep Med Rep.

[31] Feinberg I, Koresko RL, Heller N (1967). EEG sleep patterns as a function of normal and pathological aging in man. J Psychiatr Res.

[32] Ringli M (2011). The sleep EEG in children with ADHD: preliminary findings and implications for brain maturation. J Sleep Res.

[33] Winsler A, Deutsch A, Vorona RD, Payne PA, Szklo-Coxe M (2015). Sleepless in Fairfax: the difference one more hour of sleep can make for teen hopelessness, suicidal ideation, and substance use. J Youth Adolesc.

[34] Chauvette S, Seigneur J, Timofeev I (2012). Sleep oscillations in the thalamocortical system induce long-term neuronal plasticity. Neuron.

[35] Iglowstein I, Latal Hajnal B, Molinari L, Largo RH, Jenni OG (2006). Sleep behaviour in preterm children from birth to age 10 years: a longitudinal study. Acta Paediatr.

[36] Gruber R, Wise MS (2016). Sleep spindle characteristics in children with neurodevelopmental disorders and their relation to cognition. Neural Plast.

[37] Tarokh L, Saletin JM, Carskadon MA (2016). Sleep in adolescence: physiology, cognition and mental health. Neurosci Biobehav Rev.

[38] Navarrete M, Valderrama M, Lewis PA (2020). The role of slow-wave sleep rhythms in the cortical-hippocampal loop for memory consolidation. Curr Opin Behav.

[39] Li W, Ma L, Yang G, Gan WB (2017). REM sleep selectively prunes and maintains new synapses in development and learning. Nat Neurosci.

[40] Siegel JM (2011). The REM sleep-memory consolidation hypothesis. Science.

[41] Diering GH, Nirujogi RS, Roth RH, Worley PF, Pandey A, Huganir RL (2017). Homer1a drives homeostatic scaling-down of excitatory synapses during sleep. Science.

[42] Zhang J, Paksarian D, Lamers F, Hickie IB, He J, Merikangas KR (2017). Sleep patterns and mental health correlates in US adolescents. J Pediatr.

[43] Telzer EH, Goldenberg D, Fuligni AJ, Lieberman MD, Gálvan A (2015). Sleep variability in adolescence is associated with altered brain development. Dev Cogn Neurosci.

[44] Wolfson AR, Carskadon MA (2003). Understanding adolescents' sleep patterns and school performance: a critical appraisal. Sleep Med Rev.

[45] Bosl WJ, Tager-Flusberg H, Nelson CA (2018). EEG analytics for early detection of autism spectrum disorder: a data-driven approach. Scientific reports.

[46] Alfonsi V, Scarpelli S, D'Atri A, Stella G, De Gennaro L (2020). Later school start time: the impact of sleep on academic performance and health in the adolescent population. Int J Environ Res Public Health.

[47] Rigney G, Blunden S, Maher C, Dollman J, Parvazian S, Matricciani L, Olds T (2015). Can a school-based sleep education programme improve sleep knowledge, hygiene and behaviours using a randomised controlled trial. Sleep Med.

[48] Irish LA, Kline CE, Gunn HE, Buysse DJ, Hall MH (2015). The role of sleep hygiene in promoting public health: a review of empirical evidence. Sleep Med Rev.

[49] Hershner SD, Chervin RD (2014). Causes and consequences of sleepiness among college students. Nat Sci Sleep.

[50] Hale L, Troxel W, Buysse DJ (2020). Sleep health: an opportunity for public health to address health equity. Annu Rev Public Health.

[51] Buysse DJ (2014). Sleep health: can we define it? Does it matter?. Sleep.

[52] Pine AE, Liu Q, Abitante G, Sutherland S, Garber J (2022). Predictors of sleep-problem trajectories across adolescence. Res Child Adolesc Psychopathol.

[53] Lund HG, Reider BD, Whiting AB, Prichard JR (2010). Sleep patterns and predictors of disturbed sleep in a large population of college students. J Adolesc Health.

[54] Hein M, Mungo A, Hubain P, Loas G (2020). Excessive daytime sleepiness in adolescents: current treatment strategies. Sleep Sci.

[55] John B, Bellipady SS, Bhat SU (2016). Sleep promotion program for improving sleep behaviors in adolescents: a randomized controlled pilot study. Scientifica (Cairo).

[56] Tagaya H, Uchiyama M, Ohida T (2004). Sleep habits and factors associated with short sleep duration among Japanese high-school students: a community study. Sleep Biol Rhythms.

[57] Moseley L, Gradisar M (2009). Evaluation of a school-based intervention for adolescent sleep problems. Sleep.

[58] Illingworth G, Sharman R, Harvey CJ, Foster RG, Espie CA (2020). The Teensleep study: the effectiveness of a school-based sleep education programme at improving early adolescent sleep. Sleep Med X.

